# The Cortisol Paradox of Trauma-Related Disorders: Lower Phasic Responses but Higher Tonic Levels of Cortisol Are Associated with Sexual Abuse in Childhood

**DOI:** 10.1371/journal.pone.0136921

**Published:** 2015-08-28

**Authors:** Inga Schalinski, Thomas Elbert, Susann Steudte-Schmiedgen, Clemens Kirschbaum

**Affiliations:** 1 Clinical Psychology, Department of Psychology, University of Konstanz, Konstanz, Germany; 2 Institute of Biological Psychology, Technische Universität Dresden, Dresden, Germany; Central Institute of Mental Health, GERMANY

## Abstract

**Objectives:**

Inconsistent findings exist for the activity of the hypothalamic-pituitary-adrenal (HPA) axis in patients with stress related disorders. Recent studies point towards early life stress as a potential modulator.

**Methods:**

We investigated the impact of childhood sexual abuse on phasic (saliva cortisol reactivity) and tonic (hair cortisol) regulation. Furthermore, we assessed predictors on cortisol accumulation in hair. Women (*N* = 43) with stress-related disorders underwent a standardized assessment of idiographic adverse and traumatic experiences and psychopathology, while measuring salivary cortisol and, heart rate and blood pressure.

**Results:**

Comparing women with and without childhood sexual abuse revealed lower rates of responders and distinct levels of salivary cortisol to the interview in conjunction with a lower heart rate for the abused group. Childhood adversities, traumatic experiences, and depression contributed to higher hair cortisol levels.

**Conclusions:**

Our finding of lower response rate and distinct salivary cortisol pattern in individuals with childhood sexual abuse compared to individuals without early sexual abuse supports the role of environmental programming for the HPA axis. Both, childhood adversities and traumatic stress emerge as crucial factors for long-term cortisol secretion. Lower or suppressed phasic cortisol responses to trauma-related stimuli may therefore be associated with higher tonic values. Thus, early exposure to adversities may result in a biological distinct phenotype in adult patients with stress-related disorders.

## Introduction

Individuals who present with a trauma spectrum disorder frequently have an altered regulation of the hypothalamic-pituitary-adrenal (HPA) axis (see for a meta-analysis and reviews [1,2,3,4]). It is, however, not clear whether the neuroendocrine phenotype is a part of a core disease or whether it is related to environmental stressors such as childhood adversities or traumatic experiences. The most often studied hormone of the HPA axis is cortisol. Whereas a tremendous amount of studies measured salivary cortisol reactivity (with pharmacological or non-pharmacological challenge tests [5]) that reflects phasic HPA axis activity within 10–60 minutes [6], other studies measured salivary cortisol under basal conditions e.g., diurnal cortisol patterns [7]. Given the high state dependent reactivity of salivary cortisol (not only towards the experimental stressor), recent studies achieved to measure cortisol deposited in scalp hair across one to several months e.g., [8]. Here, several studies emphasize the great utility of this method for elucidating knowledge on long-term cortisol secretion in stress-related and clinical populations [8].

However, the distinct cortisol measures yielded inconsistent results for patients with posttraumatic stress disorder (PTSD) and depression [1,2,3,4]. In the vast majority of studies, some factors have drawn particular interest, including early life stress, traumatic experiences across the lifetime, the nature of trauma and the time-depending modulations [1,4]. For instance, a study found enhanced cortisol release when depressive women were facing a psychosocial stress task [9]. From an evolutionary perspective, the early environment may play an important role in programming the adult function of the HPA axis. If a developing individual is exposed to a hostile environment, adaptations of the endocrine, behavioral and autonomic nervous system would be important in order to enhance survival [10]. Supporting this hypothesis, early life stress in rodents showed enduring effects on the genome and distinct reactivity of the HPA axis that last into adulthood [11]. In postmortem brains of suicide victims, the reduced expression of the glucocorticoid receptor was also related to childhood adversities [12].

Besides patients with depression, heightened reactivity of the HPA axis towards stressful cognitive challenge or when confronted with trauma-reminders was reported for patients with PTSD related to childhood abuse and maltreatment [13,14]. However, other studies found blunted cortisol reactivity in response to psychosocial stressors for patients with early life stress [15,16], demonstrating as well a lower rate of individuals that show a cortisol increase facing the Trier Social Stress Test compared to healthy controls [16]. Distinct cortisol reactivity pattern could also be observed in women without current or past psychopathology facing public speaking and mental arithmetic stressors [17] and may relate to the trauma exposure in general rather than symptom levels. In contrast to the large amount of findings regarding salivary cortisol reactivity, hair cortisol levels in relation to early life stress were reported only in a few studies. Whereas the severity of childhood adversities showed no significant association with hair cortisol [18], a negative relationship between early life stress and hair cortisol was found for depressive patients and healthy controls [19]. In addition, a dose-dependent effect of different traumatic experiences across the lifetime on hair cortisol concentration has been reported in severely traumatized Ugandan ex-combatants, showing higher levels of hair cortisol concentrations with increasing traumatic load [20]. In contrast, the relationship in a sample of German patients with PTSD showed the reverse pattern: the higher the traumatic stress load, the lower the cortisol levels in hair [18,21]. The authors suggested that the time passed since the traumatic stress might account for the different direction of association observed across both studies. While the Ugandan sample had been exposed more continuously and more recently to traumatic stress, most of the individuals of the German sample reported more isolated traumatic experiences, more than five years prior to the investigation.

Finally, giving the above contradictory findings on cortisol activity by using short and long-term cortisol assessment approaches, there is a need for a differential theoretical framework to incorporate the distinct findings. Besides the early timing of stressful experiences, the specific nature of stress could elicit qualitatively different physiological and hormonal responses as well as distinct emotional responses [22,23]. The evolutionary based defense cascade model by Schauer and Elbert [24] provides a model that integrates differential responses to traumatic stressors. Especially threatening experiences with high proximity to an offender such as sexual and physical assaults may result in fright-flag-faint responses that differ from flight-fight responses in terms of distinct physiological as well as emotional and hormonal responses. Both types of responses are adaptive to stressful environments [10]. In humans, Griffin an colleagues [25] examined a group of female rape survivors and found a reduced heart rate and skin conductance during the assessment of the individual traumatic experiences. When the individual was exposed to bodily fluids such as sperm or blood, the cortisol response might differ due to distinct demands of the immune system e.g., [26,27]. Gola and colleagues found distinct cortisol responding during an interview of traumatic experiences in survivors of torture with and without rape experiences [28]. Whereas an adult more likely possesses the strength or the power for flight or fight, a child is more likely to show fright-flag-faint responses especially when exposed to maximal proximity such as penile penetration [29]. The HPA axis is often considered as a general system that can be activated in response to a variety of negative situations including emotion-induction procedures [6]. According to Lang’s bio informational theory of emotion [30] different responses in PTSD are viewed as resulting from an associated memory network–the so called “fear network” that are activated by internal or external trauma reminders such as an assessment of traumatic experiences. Such a challenging test on cortisol reactivity has been applied in a previous study showing increased reactivity towards the assessment of traumatic experiences [28].

However, the role of early life stress and its interplay with later ongoing stressors has not been sufficiently studied by previous HPA axis investigations on cortisol stress reactivity and long-term cortisol secretion. To allow a differentiation of phasic and tonic levels of cortisol, the current study measured acute cortisol stress reactivity and long-term hair cortisol levels. Specifically, we investigated the impact of childhood sexual abuse (and other adverse experiences) on saliva cortisol reactivity and autonomic parameters towards an idiographic assessment of traumatic experiences in female patients with stress-related psychopathology. It remains unclear whether the scars of early adverse experiences can be observed in a sample of patients with multiple exposures to trauma. If so, we would assume to find a distinct pattern of phase reactivity towards stressful interview for the subset of individuals with and without a history of childhood sexual abuse. In addition, those groups might differ as well in the response rate showing differential frequencies of baseline-to-peak increases to the trauma assessment. Further, we investigated the impact of early adverse experiences and lifetime traumatic experiences, the time elapsed since the last traumatic event as well as psychopathology on cumulative cortisol levels (in hair). In addition, we examined the association between phasic and tonic cortisol levels.

## Materials and Methods

### Ethics Statement

Prior to the interview, a comprehensive explanation of the study was provided and after written informed consent was obtained from the participants. In cases in which the participant was underaged, written informed consent was also obtained from an adult caretaker. The project and the consent form has been reviewed and specifically approved by the Institutional Review Board (Ethics Committee) of the University of Konstanz. The procedure contributing to the project complies with the ethical standards of the Helsinki Declaration of 1975, as revised in 2008.

### Participants

Patients with stress-related disorders (*N* = 43) were recruited through the outpatient clinic for refugees at University of Konstanz. To compare results of hair cortisol levels, we further recruited 12 healthy control subjects from the general community. Forty-three female survivors of violence participated in the study. The age of the participants ranged from 16 to 56 years (*M* = 34.9, *SD* = 10.5). The participants of the patient group were refugees from different regions of origin (22 Middle/ Far East, 14 Africa, 5 Balkan and 2 India) with multiple traumatic experiences across the lifespan. Four out of 43 participants smoked regularly cigarettes, and 14% took at least one psychoactive medication (*n* = 6 antidepressant and 2 out of the 6 were treated additionally with neuroleptic medication). Forty-one percent took non-prescription pain relievers (*n* = 18). Healthy subjects had similar ethnic backgrounds (5 Middle/ Far East, 5 Africa, 1 Balkan and 1 India), *χ* ^*2*^(3) = 0.64, *p* = .887, and were on average *M* = 31.9 years old (*SD* = 7.5). Three out of 12 participants smoked regularly cigarettes, but none of the participants of the healthy control group were taking psychoactive medication at the time of testing. None of the participants took medication targeting the cardiovascular system such as digitalis, beta-blockers or anticholinergics.

### Procedure and Instruments

**Fig 1:**
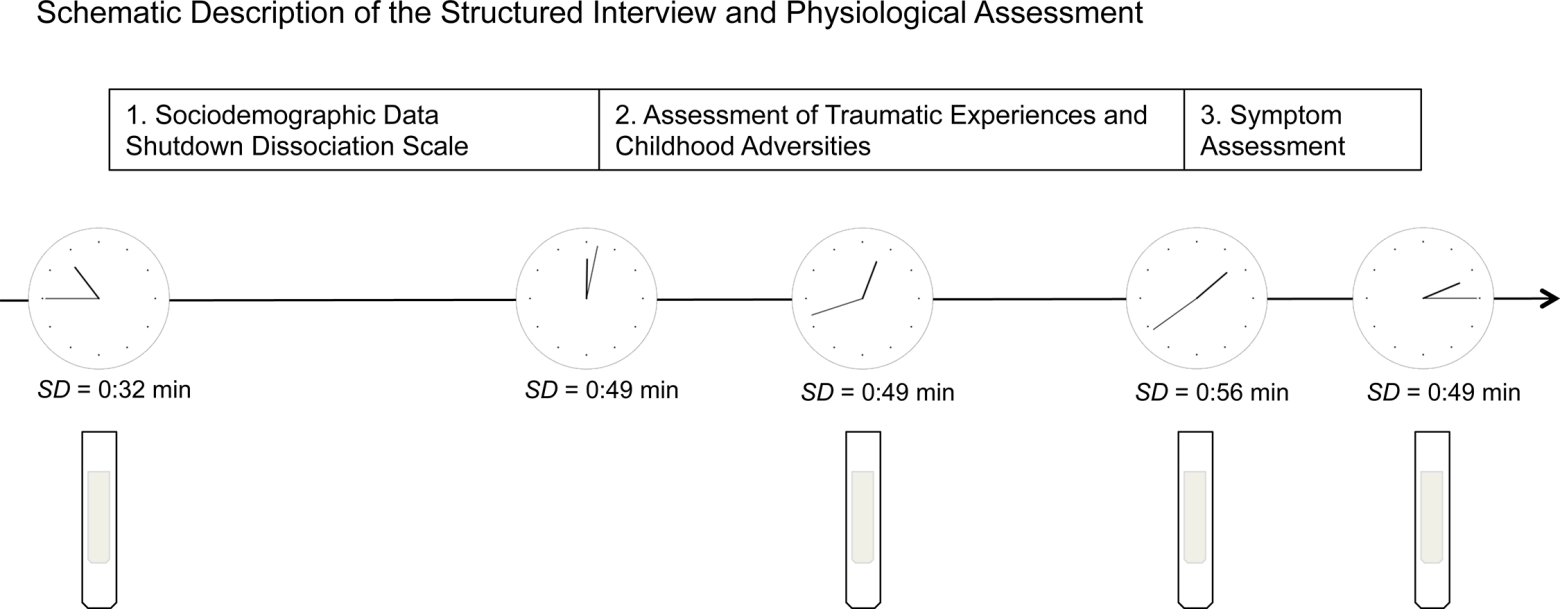
Schematic Description of the Interview Design.

The average wake-up-time was 6:20 hours (*SD* = 1:28). Participants were instructed to eat breakfast, however food, nicotine, caffeine intakes were omitted at least one-hour prior to the interview. Interviews with the patient sample started at 10:00 a.m. Salivary cortisol, heart rate, systolic and diastolic blood pressure were recorded at 4 different time points during a standardized interview. The design has been applied in a previous study [28]. The interviews were administered by experienced psychologists and trained translators. After providing written informed consent, patients gave the first saliva sample (on average at 10:45 hours (*SD* = 0:32)) and assessment of heart rate, as well as systolic and diastolic blood pressure (Fig 1). Subsequently, sociodemographic data and symptoms of shutdown dissociation were assessed [31]. The trauma assessment took on average *M* = 1:38 hours (*SD* = 0:31). The interview about traumatic experiences included (1) the event checklist [32], (2) followed by the interview of childhood adversities. The Childhood adversities up to age 18 were recorded using the Early Trauma Inventory (ETI); German version [33,34]). For the two underaged participants we adjusted the maximum age to the respective age of the participants). In an interview 55 items that score on four domains of maltreatment and abuse were assessed (general traumatic experiences, emotional abuse and neglect, physical maltreatment and sexual abuse). Any recollected experiences were accounted as one of it was experienced at least once during childhood (first 18 years of life). The sum score of all items was used to quantify the diversity of early life stress. Forty minutes after the start of the trauma assessment a second saliva sample and physiological data were obtained. In the end of the trauma assessment, a third sample and physiological data were collected. In the third part of the interview, symptoms were assessed: Symptom severity and diagnosis of PTSD were recorded with the Clinician-Administered PTSD Scale (CAPS; [35]). Current comorbid DSM-IV disorders (such as depression, dysthymia, abuse or dependency of alcohol/ illegal substances, suicidality and psychotic disorders) were assessed with the MINI International Neuropsychiatric Interviews (M.I.N.I.; [36]). The sum score of the clinician-administered Hamilton Rating Scale estimated the severity of depression (HAM-D; [37]). In the end of the interview a forth saliva sample was collected and physiological assessment was recorded. Healthy controls were interviewed following the same protocol, but did not provide salivary cortisol samples or physiological data due to shorter interview duration.

### Salivary and Hair Cortisol

Salivary cortisol samples were obtained using a collection device (Salivette, Sarstedt, Nümbrecht, Germany). After collection of samples, the device was centrifuged for 3 minutes (1400 x gravitation), and stored at -80°C until assayed. Salivary cortisol levels were measured using a commercial enzyme-linked immunosorbent assay (Cortisol ELISA, IBL International, Hamburg, Germany) according to the manufacturer’s instructions. Cross-reactivity of the anti-cortisol antibody with other relevant steroids was 7.0% (11 deoxycortisol), 4.2% (cortison), 1.4% (corticosterone), 0.35% (progesterone), and < 0.01% (testosterone, estrone, estradiol, estriol). Intra- and interassay variances were 4.8% and 5.9%, respectively. The detection limit was 0.138 nmol/l. We collected saliva samples from 40 patients. We have obtained *n* = 33 complete (with 4 saliva cortisol) samples. To distinguish between stress responder and nonresponder we calculated the rate of responders defined by a baseline-to-peak increase of 1.5 nmol/l [38]. The area under the curve with respect to ground (AUC_G_) as well as the area under the curve in respect to increase (AUC_I_) was calculated as a composite measure according to Pruessner and colleagues [39].

Two 3 cm segments of hair (in case of individuals with sufficient hair length) were cut scalp-near from a posterior vertex position. Using the protocol of Stalder and Kirschbaum [8], 5 mg hair samples were washed with 2.5 mL isopropanol, and cortisol was extracted with 1800 mL methanol for 18 hours at 45°C. Cortisol levels were measured in the resuspended extracts by a commercial immunoassay with chemilumincescence detection (CLIA, IBL-Hamburg, Germany).

### Assessment of Heart Rate, Systolic and Diastolic Blood Pressure

Heart rate and blood pressure was measured by a semi-automatically sphygmomanometer (Boso Medicus, Bosch and Sohn, Jungingen, Germany). The device’s blood pressure measurements range from 40–280 mmHg (accuracy of ± 3 mmHg) and for heart rate from 40 to 200 beats per minute (accuracy ± 5%). The cuff was placed above the elbow on the brachial artery on the upper arm at the heart level. The measurement took place in sitting position and was initiated by the interviewer.

### Statistical Analysis and Data Exclusion

Analyses were performed using R version 2.15.1 and SPSS 21. The significance level was set at .05. Demographic as well as clinical data were compared with a one-way ANOVA. Salivary cortisol values were transformed using natural log-transformation to reduce positive skewness. Samples with insufficient hair (less than 3.8 mg/ segment) and high outlying values of 3 SD above the mean (*n* = 1) were excluded from further analyses. Because of hair length (less than 6 cm), the second segment could not be analyzed from *n* = 14 participants. Further, cumulative cortisol secretion differed between African-textured hair samples and other hair samples (first segment: *Mdn*_African_ = 21.1; *Mdn*_Other_ = 8.5). Differences have been reported in another multi-ethnic study [40], that might be due to differences in hair growth and hair care [41,42]. For the correlational analysis, we controlled for the effect of the variable “African-textured hair” reporting partial correlation for the first segment. To reduce positive skewness, we applied natural log-transformations to the hair cortisol values. To analyze the cortisol as well as physiological reactivity, we applied repeated measures ANOVA using time as a repeating factor (baseline, 40 minutes after the onset, and twice after idiographic assessment of traumatic experiences) and group (with and without childhood sexual abuse). For violation of the assumption of sphericity, degrees of freedom were adjusted using the Greenhouse-Geisser estimates of sphericity, for repeated measures of salivary response: ε = .76, and for repeated measures of heart rate: ε = .80. Comparing the patient sample of those with and those without childhood sexual abuse revealed also significant differences in all other types of childhood adversities: the patient group with childhood sexual abuse reported more general traumatic experiences in childhood (*t*(41) = -2.49, *p* = .017), more experiences of physical maltreatment (*t*(41) = -3.26, *p* = .002), and more emotional abuse (*t*(41) = -3.51, *p* = .001) compared to the non-abused patient group (Table 1). Missing saliva samples as well as physiological data were excluded from repeated measures analysis of variance (ANOVA), but considered in post-hoc *t*-test comparisons with Bonferroni adjusted significance threshold (0.05/ number of tests) as well as in presented data. For descriptive purposes, mean data in figures are presented in original units. The independent impact of the following variables on the hair cortisol concentration was evaluated using conditioned random forest regression within the patient group (‘cforest’ in R package party). The following variables were entered in the model: number of hair washes per week, severity of childhood adversities, number of different traumatic event types across life time, symptom severities of PTSD, depression and shutdown dissociation, time since the last traumatic event as well as the binary variable African-textured hair. A variant of Breiman’s approach was applied and generated 500 conditional trees using three variables randomly selected at each node. All variables were entered into a conditional model for predicting the hair cortisol concentration. With an iterative procedure, reparametrization of variable importance was performed assigning best predictors the highest value, the second best the second highest and so on. Non predicting variables with importance < 0, were coded with 0. The area under the curve with respect to ground (AUC_G_) as well as the area under the curve in respect to increase (AUC_I_) was calculated as a composite measure according to Pruessner and colleagues [39], and related to hair cortisol levels.

**Table 1:** Demographic Data, Hair-related Variables and Clinical Data for the Patient Group and the Healthy Control Group. Results of Saliva Cortisol and Heart Rate.

	Healthy Control Group *n* = 12	No Childhood Sexual Abuse *n* = 22	Childhood Sexual Abuse *n* = 21	Group Comparisons
**Personal Data**				
Age (in Years)	31.9 (7.5)	32.6 (9.0)	37.2 (11.6)	*F*(2,52) = 1.62, *p* = .208
Body Mass Index	23.3 (4.8)	26.0 (5)	26.7 (6.4)	*F*(2,50) = 1.45, *p* = .244
Use of Oral Contraception (1 = yes, 0 = no)	1 (8.3%)	0	0	*χ²*(2) = 3.32, *p* = .191
Regular Medication (1 = yes, 0 = no)	1 (8.3%)	12 (54.5%)	8 (38.1%)	*χ²*(2) = 10.03, *p* = .007
Active Smoking (1 = yes, 0 = no)	3 (25%)	2 (9.1%)	2 (9.5%)	*χ²*(2) = 1.69, *p* = .430
Regions of Origin N, %				*χ²*(6) = 2.90, *p* = .822
Middle and Far East	5 (41.6%)	11 (50%)	11 (52.4%)	
The Balkans	1 (8.3%)	3 (13.6%)	2 (9.5%)	
Africa	5 (41.6%)	6 (27.3%)	8 (38.1%)	
India	1 (8.3%)	2 (9.1%)	0	
**Hair-Related Variables** ^1^				
Hair Washes/ Week^1^	4.1 (2.7)	2.5 (1.6)	2.5 (1.2)	*F*(2,48) = 3.47, *p* = .039
Curls or Waves (1 = yes, 0 = no) ^1^	9 (75%)	15 (78.9%)	17 (89.5%)	*χ²*(2) = 0.52, *p* = .773
Semi-permanent Color/ Coloration (1 = yes, 0 = no) ^1^	3 (25%)	7 (31.8%)	9 (42.8%)	*χ²*(2) = 1.33, *p* = .515
**Traumatic Experiences and Adverse Childhood Experiences**				
ETI Sum Score	5.9 (4.7)	7.6 (6.3)	18.6 (9.1)	*F*(2,52) = 16.98, *p* < .001
ETI General Trauma	2.1 (1.7)	3.7 (3.2)	6.1 (3.0)	*F*(2,52) = 8.11, *p* = .001
ETI Physical Punishment	1.6 (1.4)	1.8 (2.0)	4.0 (2.4)	*F*(2,52) = 7.91, *p* = .001
ETI Emotional Abuse	1.9 (2.4)	2.1 (2.9)	5.2 (2.8)	*F*(2,52) = 8.37, *p* = .001
ETI Sexual Abuse	0.3 (0.7)	0 (0)	3.3 (2.7)	*F*(2,52) = 22.91, *p* < .001
Number of Different Traumatic Event Types	2.6 (1.6)	7.6 (2.9)	9.2 (3.4)	*F*(2,51) = 19.99, *p* < .001
Time Elapsed since Last Traumatic Event (in years) ^1^	7.3 (5.9)	3.3 (3.1)	4.7 (6.0)	*F*(2,47) = 1.90, *p* = .160
**Symptom Severity**				
PTSD Symptom Severity	0.7 (2.3)	75.5 (27.3)	73.8 (26.5)	*F*(2,52) = 44.99, *p* < .001
Hamilton Depression Score	2.8 (2.7)	18.6 (9.2)	18.3 (8.6)	*F*(2,52) = 17.98, *p* < .001
Shutdown Dissociation Score	1.0 (1.3)	16.7 (8.7)	15.8 (8.9)	*F*(2,52) = 17.77, *p* < .001
**Salivary Cortisol (natural log-transformed)**				
At Baseline		2.06 (0.4)	2.08 (0.38)	*t*(39) = -0.19, *p* = .852, CI_95_[-0.27, 0.23]
40 Minutes of Trauma Assessment		2.10 (0.61)	1.65 (0.32)	*t*(22.55) = 2.58, *p* = .017, CI_95_[0.1, 0.79]
At the End of the Trauma Assessment		2.12 (0.6)	1.63 (0.34)	*t*(25.84) = 2.89, *p* = .008, CI_95_[0.14, 0.85]
At the End of the Interview		1.99 (0.53)	1.78 (0.51)	*t*(35) = 1.24, *p* = .224, CI_95_[-0.13, 0.56]
**Heart Rate (in beats per minute)**				
At Baseline		80.8 (12.6)	75.7 (9.5)	*t*(39) = 1.47, *p* = .150, CI_95_[-1.9, 12.1]
40 Minutes of Trauma Assessment		79.1 (10.8)	70.5 (8.3)	*t*(37) = 2.79, *p* = .008, CI_95_[2.4, 14.9]
At the End of the Trauma Assessment		77.9 (11.3)	69.5 (7.4)	*t*(23.7) = 2.45, *p* = .022, CI_95_[1.3, 15.5]
At the End of the Interview		77.9 (11.3)	70.7 (8.7)	*t*(34) = 2.16, *p* = .038, CI_95_[0.4, 14]

**Note.** PTSD = posttraumatic stress disorder; ETI = Early Trauma Inventory

^1^related to the total number of hair samples, *n* = 19 for the group without childhood sexual abuse and *n* = 19 for the group with sexual abuse in childhood.

^2^healthy controls without exposure to traumatic events were excluded from the analysis

## Results

### Clinical Characteristics

Neither the age, nor the body mass index, nor the ethnical background differed between groups all *ps* > .2 (Table 1). Out of 43 patients, 36 fulfilled all DSM-IV criteria of PTSD. Those 36 suffered additionally from comorbid depression. Four out of 43 had partial symptoms of PTSD (including re-experiencing symptoms, but failed to reach Criteria C), and three fulfilled the criteria for depression. Three out of 43 showed depression without any signs of current PTSD re-experiencing symptoms. The following traumatic event types were reported most frequently in the patient sample: physical assault with life threat (72%), assault with a weapon (62%) and sexual assault (58%). The healthy control group reported on average *M* = 2.6 (*SD* = 1.6) different traumatic event types across the life span. Only 2 out of 12 controls were unexposed to traumatic stress.

### Phasic Cortisol in Saliva

**Fig 2:**
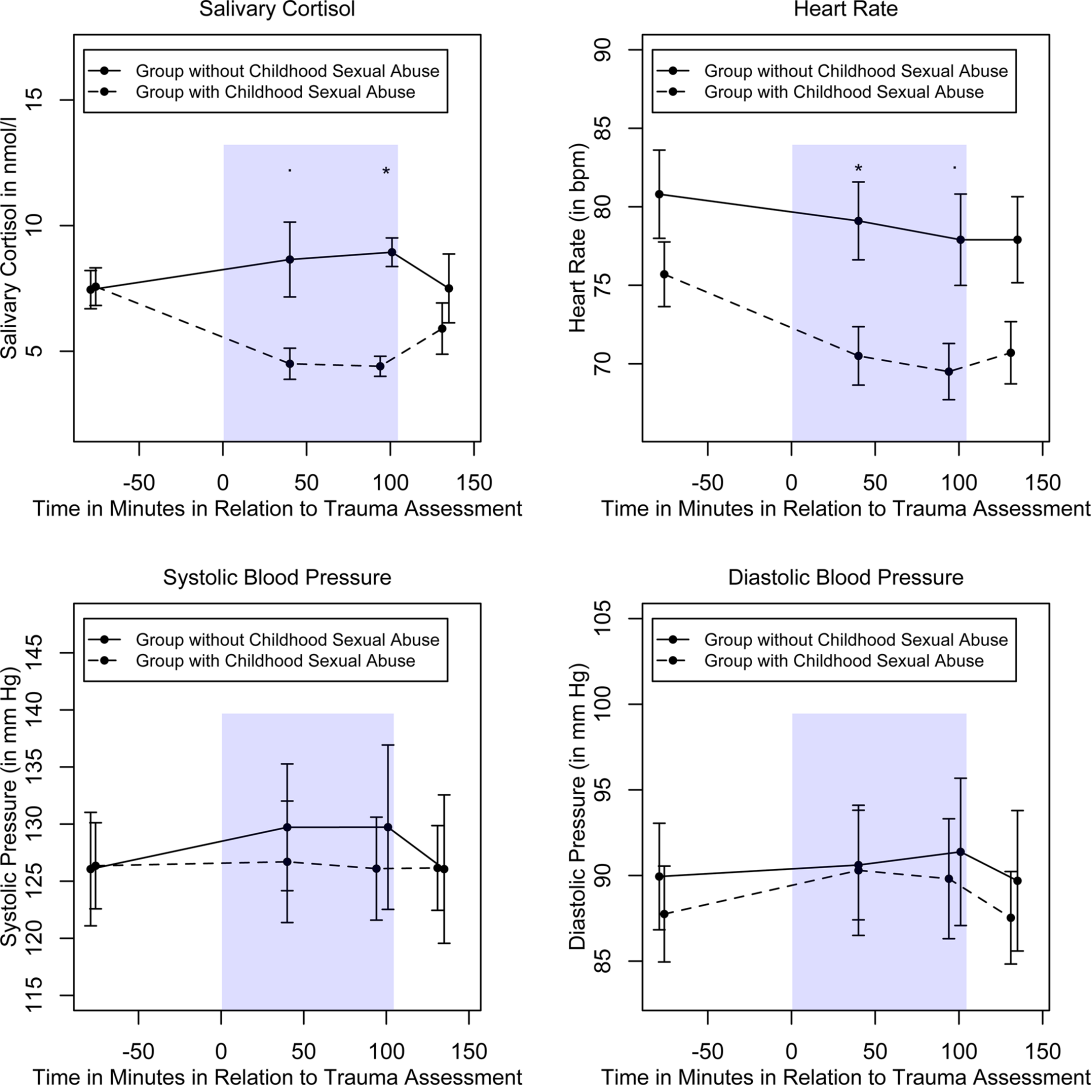
Salivary Cortisol, Heart Rate and Blood Pressure across the Interview as a Function of Group (with and without Childhood Sexual Abuse). The mean reactivity of saliva cortisol (in original non-log-transformed units), heart rate and blood pressure response separately for the group with and without childhood sexual abuse. The error bars indicate the standard error of the mean (*SEM*). The blue-shaded area marks the average time of trauma assessment. “*” indicates significant group difference at *p* ≤ .050, and “·” at *p* ≤ .10 (with Bonferroni correction)

Fig 2 illustrates the mean salivary cortisol response to the idiographic trauma interview. Salivary cortisol levels differed along the interview (*F*(2.29, 71) = 4.06, *p* = .017, *η* ^2^ = .12), as well as for the group with and without childhood sexual abuse (*F*(1, 31) = 6.25, *p* = .018, *η* ^2^ = .17). Interestingly, the ANOVA showed a significant time x group interaction (*F*(2.29, 71) = 3.08, *p* = .045, *η* ^2^ = .09). Whereas the group with childhood sexual abuse responded with a down regulation of cortisol, the group without childhood sexual abuse did not differ from baseline. Post-hoc *t*-tests indicated a trend of a group effect at the time point “40 minutes of trauma assessment” (*t*(22.55) = 2.58, *p* = .017, Cohen’s *d* = 0.92). At the end of the trauma assessment, there was a significant group effect showing lower cortisol levels for those with childhood sexual abuse compared to those without early sexual abuse (*t*(23.65) = 2.89, *p* = .008, Cohen’s *d* = 1.0). After including covariates in the repeated ANOVA (number of traumatic experiences, PTSD, depression and shutdown dissociation severity, age, wake up time, duration of sleep), the group as well as the interaction effect remained statistically significant. Additionally, the pattern of main results was confirmed in analyses excluding data from patients without PTSD.

Additional analysis yielded a response rate (% of those showing a baseline-to-peak increase of 1.5 nmol/l at any time across the interview) of 42.4% facing the trauma assessment (56.3% in the non-abused group versus 20.8% in the sexually abused group). Comparing both groups at the different time points of assessment showed lower response rates for those with reported childhood sexual abuse compared to non-abused (at 40 minutes of trauma assessment: 12.5% versus 36.4%, *χ* ^*2*^(1) = 3.14, *p* = .077; at the end of the trauma assessment: 8.3% versus 36.4%, *χ* ^*2*^(1) = 5.1, *p* = .024 and at the end of the interview: 12.5% versus 22.7%, *χ* ^*2*^(1) = 0.78, *p* = .376).

### Heart Rate and Blood Pressure Responses to Idiographic Trauma Interview

Similarly to the cortisol response, the heart rate showed a decrease along the interview for the group that reported childhood sexual abuse compared to the non-abused group, yielding in a significant main effect of group (*F*(1, 28) = 5.55, *p* = .026, *η* ^*2*^ = .17). Further, there was a significant main effect of time (*F*(2.4, 67.26) = 4.75, *p* = .008, *η* ^*2*^ = .15), however the interaction of time and group did not reach significance (*F*(2.4, 67.26) = 2.04, *p* = .129, *η* ^*2*^ = .07). Post-hoc *t*-tests revealed a significant difference “at 40 minutes of idiographic trauma interview”, (*t*(37) = 2.79, *p* = .008, Cohen’s *d* = 0.89), and a trend of significance at the time point “at the end of the trauma assessment” (*t*(23.7) = 2.45, *p* = .022, Cohen’s *d* = 0.88).

In contrast, the analysis showed no significant reactivity along the interview for systolic blood pressure (*F*(3, 87) = 0.87, *p* = .461, *η* ^*2*^ = .03), no interaction (*F*(3, 87) = 0.23, *p* = .876, *η* ^*2*^ < .01) and no group effect (*F*(1, 29) = 0.09, *p* = .768, *η* ^*2*^ < .01).

Neither the diastolic blood pressure varied along the interview (*F*(3, 87) = 0.82, *p* = .489, *η* ^*2*^ = .03), nor the interaction effect of time and group reached significance (*F*(3, 87) = 1.09, *p* = .360, *η* ^*2*^ = .04), nor the group effect (*F*(1, 29) = 0.03, *p* = .873, *η* ^*2*^ < .01, Fig 2).

### Cortisol Levels in Hair

There were significant group differences in the number of hair washes per week where the healthy control group exhibited the following values (*M* = 4.1, *SD* = 2.7) when compared to the patient groups at (*M* = 2.5, *SD* = 1.5). This variable was then considered as a covariate in the repeated ANOVA measurements (compare Table 1) where we accounted for the main effect of hair washes per week to analyze the hair cortisol levels as a function of group. For the first segment, the log-transformed cortisol concentration in hair did not differ significantly between the groups (*M*_*control*_ = 2.42, *SD* = 0.9, 95% CI[1.94, 2.93], *M*_*patient*_ = 2.44, *SD* = 0.78, 95% CI[2.2, 2.69]) with *F*(1, 48) = 0.22, *p* = .645, *η* ^*2*^ < .01, when the effect of the covariate frequency of hair washes was considered, *F*(1, 48) = 2.39, *p* = .129, *η* ^*2*^ = .05. Whereas the healthy control group had on average a cortisol concentration (log-transformed) of *M* = 0.84 (*SD* = 0.83, 95% CI[0.16, 2.23]), the patient group obtained on average higher values in the second segment *M* = 1.79 (*SD* = 1.01, 95% CI[1.45, 2.17]). However, this difference was not significant (*F*(1, 35) = 2.91, *p* = .097, *η* ^*2*^ = .08), when considering the effect of the covariate (*F*(1, 35) = 0.7, *p* = .800, *η* ^*2*^ < .01).

### Correlates for Tonic Cortisol Levels in Hair within the Patient Sample

A positive correlation was found for the severity of childhood adversities and cortisol accumulated in hair after controlling for the variable African-textured hair (first segment: *pr* = .38, *p* = .019; second segment: *r* = .43, *p* = .016). In particular, the subscale sexual abuse in childhood showed a positive relationship with hair cortisol levels (first segment: *pr* = .46, *p* = .004; second segment: *r* = .47, *p* = .008). Moreover, the number of different traumatic event types was also positively associated with hair cortisol concentrations (first segment: *pr* = .45, *p* = .005; second segment: *r* = .43, *p* = .016). Neither the time since the last traumatic event, nor the severity of depression, nor the severity of PTSD symptoms, nor the severity of shutdown dissociation was correlated with hair cortisol levels (Table 2).

**Table 2:** Correlations between Hair Cortisol Concentrations, Severity of Childhood Adversities, Number of Traumatic Experiences and Psychopathology.

	Segment 1^1 (^ *n* = 38)		Segment 2 (*n* = 29)	
Measure	*pr*	95% CI	*r*	95% CI
ETI Sum Score	.38*	[.03, .65]	.43*	[.09, .71]
ETI General Trauma	.28	[-.04, .55]	.33	[-.09, .63]
ETI Physical Punishment	.26	[-.07, .58]	.25	[-.12, .59]
ETI Emotional Abuse	.27	[-.1, .57]	.36*	[-.01, .66]
ETI Sexual Abuse	.46*	[.04, .69]	.47*	[-.06, .74]
Number of Different Traumatic Event Types	.45*	[.17, .66]	.43*	[.07, .66]
Time since the Last Traumatic Event	.07	[-.26, .43]	.21	[-.16, .53]
PTSD Symptom Severity	.12	[-.24, .41]	-.09	[-.42, .22]
Hamilton Depression Score	.28	[.02, .51]	.14	[-.13, .38]
Shutdown Dissociation Score	.17	[-.15, .47]	.08	[-.28, .45]

* *p* ≤ .05.

*pr* = partial correlation; CI = confidence interval; ETI = Early Trauma Inventory. PTSD = Posttraumatic Stress Disorder. ^1^ partial correlation were calculated for the Segment 1, controlling for the variable African-textured Hair

### Predictors for Tonic Hair Cortisol Concentration Within the Patient Group

**Fig 3:**
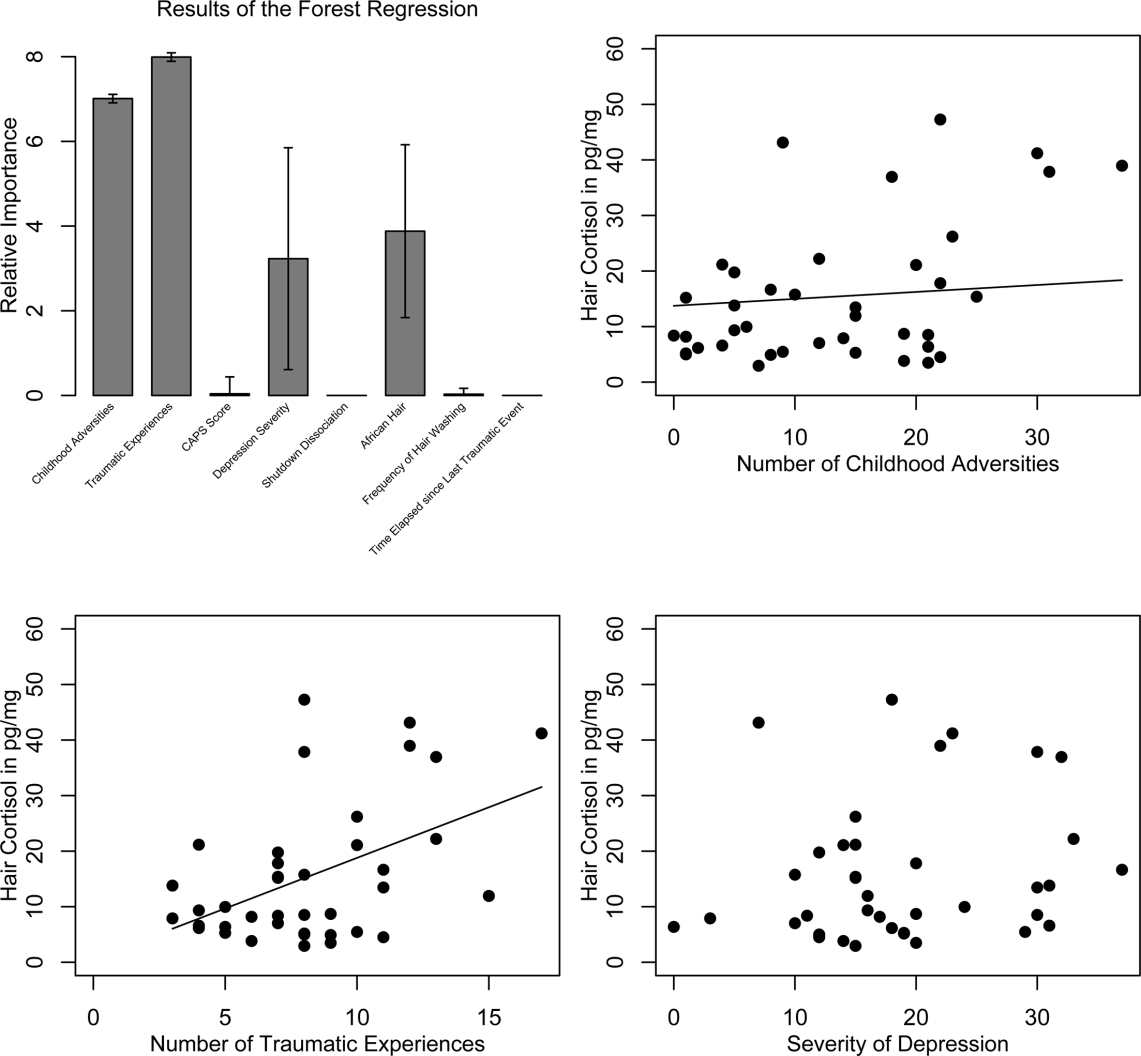
Predictors of Hair Cortisol Levels. Results of the conditioned random forest regression as well as scatterplots for each important variable. Hair cortisol levels are displayed in original units (in pg/mg). Filled circles = patient group member. CAPS = Clinician-Administered PTSD Scale.

The predictive model derived from the conditioned forest regression model indicated the impact of (1) the number of traumatic experiences, followed by (2) childhood adversities and (3) depression severity as well as the variable (4) African-textured hair (Fig 3). No meaningful contribution to hair cortisol levels was found for PTSD severity and shutdown dissociation, the time elapsed since the last traumatic experience and frequency of hair washing. To access the direction of relationship, we calculated correlation coefficients and considered the binary variable “African-textured hair” (1 = yes, 0 = no) for the first segment. There was a positive association between traumatic experiences as well as hair cortisol levels when controlling for the binary variable “African-textured hair”: *pr* = .45, *p* = .005, 95% CI [.16, .66]. In the second segment the association was replicated: *r* = .43, *p* = .016, 95% CI [.09, .65]. When considering the binary variable, the severity of childhood adversities was correlated with the hair cortisol level: *pr* = .38, *p* = .019, 95% CI [.03, .65]. Again, this association was found as well in the second segment: *r* = .43, *p* = .016, 95% CI [.02, .71]. The predictive forest regression model indicated a meaningful contribution of depression symptoms independent of the other predictors for the cumulated hair cortisol, however the correlation coefficient did not reach significance for the first segment: *pr* = .28, *p* = .090, 95% CI [.05, .50] nor for the second segment: *r* = .14, *p* = .461, 95% CI [-.14, .39].”

### Correlates for Tonic and Phasic Cortisol Levels

There was no correlation between the AUC_G_ and hair cortisol concentrations, neither for the first nor for the second segment (first segment: *pr* = -.14, *p* = .484, 95% CI[-0.43, 0.21], second segment: *r* = -.11, *p* = .648, 95% CI[-0.46, 0.33]). However, individuals with heightened salivary cortisol reactivity (AUC_I_) showed higher hair cortisol levels in the first segment (*pr* = .39, *p* = .043, 95% CI[-0.03, 0.63]), but not in the second segment (*r* = .24, *p* = .293, 95% CI[-0.2, 0.55]).

## Discussion

The aim of the study was to assess the impact of early life stress and cumulative traumatic experience on cortisol secretion (salivary cortisol and hair cortisol) that might add to heterogeneity within groups as well as adding to inconsistencies across groups. In line with earlier studies, we found evidence for a long-lasting alteration of HPA axis activity within a group of patients with trauma-related disorders e.g., [9,15,16,20]. In addition to previous reports, we found distinct activity patterns of cortisol (measured by responder rates facing the trauma assessment, mean salivary cortisol and cortisol accumulated in hair) among patients displaying hints of neurobiological subtypes. Differential HPA-reactivity phenotypes (responders and non-responders) have also been reported in a recent study in female patients with PTSD. Facing the Trier Social Stress Test, the PTSD group with early and adulthood trauma showed lower rates of responders (43.5%) compared to healthy non-exposed controls (88.9%) [16]. Besides the distinct reactivity pattern of the cortisol secretion in the current study, the group with sexual abuse (and other adverse experiences) showed lower heart rates during the trauma assessment compared to the group without early sexual abuse. The differential effects within the patient group facing idiographic trauma assessment further highlight the impact of early life stress on alternations of the HPA axis as well as the physiology. Where increased physiological responding is a robust correlate of trauma-survivors [43], some studies failed to find the exaggerated reactivity e.g., [25]. Non-responsiveness and suppressed heart rate activity as well as hyperactivity have been reported in response to script driven imagery in patients with severe PTSD [44]. In contrast to some previous results [28], but in line with Kolassa and colleagues [45], we failed to find an increase in salivary cortisol during the trauma interview [28]. However, the sample characteristics differed in terms of gender and reported types of trauma exposure. Kolassa and colleagues could not find a difference between the trauma assessment and a control condition (assessment of absorption behavior) as well as no increase of salivary cortisol in male refugees with torture experiences and PTSD [45], whereas Gola and colleagues investigated salivary cortisol mostly in male survivors with and without sexual abuse during torture (adulthood). Assuming that therapeutic variables such as establishing a supportive, empathic and controllable environment during the assessment did not systematically differ between the group with and without childhood sexual abuse, suggests that the early experiences of sexual abuse is the main factor accounting for the present findings. The specific nature of childhood sexual abuse could elicit qualitatively different emotional response, particularly shame [3], physiological (down-regulation) and endocrine patterns [15,22,23,24], and therefore confirms the hypothesis of early programming of the HPA-axis [11]. In particular high proximal traumatic experiences such as sexual abuse in the developing individual might shape the primary mode of responding. Some animal studies show the long-lasting effect of early environments on the neural circuits that control the stress response [46,47], and form an adaptive stress phenotype [10].

Not only the early environmental conditions but also subsequent traumatic stress had an impact on long-term cortisol secretion as shown in heightened cortisol levels that were accumulated in hair across months for those patients with more traumatic experiences. The forest regression model indicated the independent impact of childhood adversities, the number of different traumatic experiences, as well as depression severity within the patient group. Those patients with more childhood adversities or various types of traumatic experiences cumulated higher levels of cortisol in hair. In line with previous findings, patients that grew up in neglectful, maltreating environments were also more likely to be exposed to traumatic stressors across their lifespan (*r* = .37*, data not reported) [48]. Additional to this re-victimization bias in the sample, both variables (childhood adversities and traumatic stress load) contributed to higher long-term cortisol levels. The depression severity may add to the vulnerability of higher cortisol aggregation in hair. Although, the result of the forest regression suggests an independent effect of depression severity on hair cortisol, we failed to find a significant positive correlation. The additional effect of depression is in line with previous studies, pointing to HPA axis hyperactivity e.g., [49]. Besides the effect of depression, our results support the assumption that cortisol levels are related to trauma exposure in general rather than symptom levels [3]. Our results are in line with the findings of the Ugandan sample showing higher levels of cortisol deposited in hair with increasing trauma load [20]. Whereas the authors pointed towards the recency of traumatic stress to explain the direction of the correlation [18], our results showed that lifetime traumatic stress correlated positively with hair cortisol independent of the time elapsed since the last traumatic event. We can only speculate about the inconsistent findings. First, there might be a dose-dependent change of down- and up-regulated HPA axis activity, that follows a U-shaped relationship between traumatic stress and long-term cortisol levels: Showing a decreasing relationship with HPA axis activity following a certain range of trauma load (e.g., 1–5 different types of traumatic experiences) and showing the opposite following more repeated traumatic stress (more than 5 different types of traumatic experiences). The other explanation might be that the timing-whether the traumatic stress has occurred in vulnerable periods during childhood- has shaped the HPA axis activity [50]. The childhood reflects a particular sensitive time for the developing individual, and high exposure to stress during windows of vulnerability might have sustainable effects on the adult function of the HPA axis [4,22,51,52].

We cannot conclude whether the snap shot of salivary response is related to the long-term accumulated cortisol concentration in hair or whether the distinct direction of responses present two distinct allostatic processes. Despite the fact that both measures show opposite HPA axis pattern, but in line with a recent finding [21], the direct relationship of the composite AUC_I_ showed the opposite suggesting lower hair cortisol values for those with a higher decrease during the interview, whereas at the same time those with sexual abuse in childhood had lower salivary cortisol responses and higher levels of hair cortisol. How does the result “lower phasic response in salivary cortisol” in survivors with childhood sexual abuse as well as higher levels of other childhood adversities fit to the “positive cumulative effect in long-term cortisol”? One possible explanation could be, that there is a delayed release of cortisol as reported in a previous study with victims of childhood sexual abuse and high dissociative symptoms. With a delay of 24 hours after assessing prior traumatic experiences the group with sexual abuse showed increased activity of the HPA axis [53]. Possibly, the HPA axis is less likely reactive when confronted with an acute stressor, but shows a delayed rebound following the stressor, resulting in higher levels of cortisol aggregated into the hair. Alternatively, the short-term and long-term HPA axis activity might have common roots (environmental adversities in the early life), whereas the long-term HPA axis activity is influenced additionally by the later environmental stress (traumatic stress). Our results point towards the explanation of the impact of early life stress (indicated by the long-lasting impact of childhood adversities on distinct salivary cortisol as well as an impact of early life stress on hair cortisol level). Early life stress superimposed the effect of multiple traumatic experiences across the lifespan on HPA axis activity. Thus, early exposure to adversities may result in a biological distinct phenotype in patients with stress-related disorders. In the present study, we focused on postnatal stressors. Other studies have shown that prenatal or early postnatal stressors effect the methylation of the glucocorticoid receptor gene of the offspring [54] or HPA-axis regulating genes [16].

In contrast to previous reports, we did not find differences in the long-term cortisol levels between healthy controls and patients [18,20]. Where previous studies compared patients with traumatic experiences to healthy controls without trauma exposure, the present study lacks an unexposed control group. Group differences might be more pronounced when exposed versus non-exposed groups are compared [18], however, might as well be dependent on the trauma-related characteristics of the study sample (single-event trauma versus multiple traumatic experiences and early traumatic experiences). The results underpin as well as well the importance to take childhood adversities and traumatic experiences into account when studying cortisol in comparative designs.

One major limitation is the challenging test that was used in the present study. Compared to psychosocial stressors in healthy controls e.g., the Trier Social Stress Test [55] elicits rates of responding above 80% [38], however the response rate was lower at 42.4% for the trauma assessment in the current sample. The different responder rates could be due to the stressor itself (psychosocial versus trauma assessment) or might be specific to the study sample with multiple exposures across lifetime that might be associated with blunted reactivity [15,16]. Even though, the response rate is similar to a recent study showing as well lower responder rates (43.5%) facing the Trier Social Stress Test [16], the present findings needs further replication to separate the cortisol pattern from regular circadian salivary secretion. Future studies should assess the various impacts of stressors within the same individuals (e.g., psychosocial, cognitive challenges and trauma assessment) as well as compare the reactivity with a time-matched control condition without stress induction. Because of the lack of a control group, we cannot conclude whether the observed salivary cortisol pattern that varies with childhood sexual abuse is specific to patients with trauma-related disorders or whether it would also be present in trauma-exposed others. Another limitation is the age of the participants that ranged across four decades and might therefore contribute to the heterogeneity, i.e., reduced the effect size.

### Conclusions

We found distinct cortisol reactivity in individuals with childhood sexual abuse compared to individuals without early sexual abuse, supporting the role of environmental programming for the HPA axis early in life [13]. Further, the current finding of long-term accumulation of cortisol in hair suggests two major pathways to enhanced accumulation of cortisol: Both childhood adversities and traumatic stress emerge as crucial factors for long-term cortisol secretion. Recognition of childhood adversities within discrete diagnostic entities will expand findings (such as distinct neurobiological underpinning). The opposing results show distinct allostatic responses to acute and chronic stress.

## Supporting Information

S1 Dataset All relevant data are within the paper and its Supporting Information files. (XLSX)
